# Growth and characterization of TiO_2_ nanotubes from sputtered Ti film on Si substrate

**DOI:** 10.1186/1556-276X-7-388

**Published:** 2012-07-12

**Authors:** Karumbaiah N Chappanda, York R Smith, Swomitra K Mohanty, Loren W Rieth, Prashant Tathireddy, Mano Misra

**Affiliations:** 1 Electrical and Computer Engineering Department, University of Utah, 50 South Central Campus Dr, MEB 3280, Salt Lake City, UT, 84112, USA; 2 Metallurgical Engineering Department, University of Utah, 135 South 1460 East, WBB 00412, Salt Lake City, UT, 84112, USA; 3 Chemical Engineering Department, University of Utah, 50 South Central Campus Dr, MEB 3290, Salt Lake City, UT, 84112, USA

**Keywords:** TiO_2_ nanotubes, Si substrate, Room temperature

## Abstract

In this paper, we present the synthesis of self-organized TiO_2_ nanotube arrays formed by anodization of thin Ti film deposited on Si wafers by direct current (D.C.) sputtering. Organic electrolyte was used to demonstrate the growth of stable nanotubes at room temperature with voltages varying from 10 to 60 V (D.C.). The tubes were about 1.4 times longer than the thickness of the sputtered Ti film, showing little undesired dissolution of the metal in the electrolyte during anodization. By varying the thickness of the deposited Ti film, the length of the nanotubes could be controlled precisely irrespective of longer anodization time and/or anodization voltage. Scanning electron microscopy, atomic force microscopy, diffuse-reflectance UV–vis spectroscopy, and X-ray diffraction were used to characterize the thin film nanotubes. The tubes exhibited good adhesion to the wafer and did not peel off after annealing in air at 350 °C to form anatase TiO_2_. With TiO_2_ nanotubes on planar/stable Si substrates, one can envision their integration with the current micro-fabrication technique large-scale fabrication of TiO_2_ nanotube-based devices.

## Background

TiO_2_ is a material of great interest due its low cost, stability, and interesting electrical and optical properties [1]. TiO_2_ nanotubes (T-NT), compared to TiO_2_ films, have a large surface area-to-volume ratio. Synthesis of these tubes via electrochemical anodization has been studied extensively over the past decade [2]. Due to their interesting electrical and optical properties, TiO_2_ nanotubes are shown to have various applications such as harvesting solar energy [3], charge storage [4], and sensors [5,6]. However, these applications have been demonstrated by anodizing Ti foil, which is the limiting factor for commercialization since synthesis of T-NT on Ti foil limits the integration onto wafer-scale devices, which is required for large-scale production. Current semiconductor fabrication techniques use micro/nano-scale precision, which requires planar/stable substrates such as Si wafer for manufacturing compact and powerful devices. Commonly used titanium foils are mechanically flexible, rendering them prone to bending which inhibits the ability to achieve the micro/nano-scaled precision. Hence, stable/planar substrates are required for integrating T-NT with the current semiconductor fabrication techniques for manufacturing compact and powerful T-NT-based devices. Growth of T-NT on stable/planar substrates such as glass and Si deposited by sputtering has been previously demonstrated [7,8]. However, in their synthesis of T-NT, low temperatures (0 °C) with a maximum voltage of 35 V were used, limiting the diameter of the tubes. Most of the micro-electro-mechanical systems (MEMS) and nano-electro-mechanical systems (NEMS) require a thin non-conductive layer to electrically isolate the device from the substrate and in order to define an electrical path to the device. Similarly, it is possible that a non-conductive layer isolating the tubes from the substrate is required in T-NT-based MEMS/NEMS. The T-NT synthesized per the method presented here generates nanotubes that are isolated from the substrate, which could prove advantageous while fabricating T-NT-based devices.

Here, we present for the first time, the synthesis of T-NT from thin Ti film on Si wafer at room temperature and at high anodization potentials up to 60 V. Direct current (D.C.)-sputtered thin Ti film, along with an organic electrolyte with approximately 6.5 pH, was used for the synthesis of the tubes. A 100-nm-thick layer of thermally grown SiO_2_ was used to electrically isolate the synthesized T-NT to demonstrate one of the suitability of the tubes for integration with the current MEMS/NEMS devices. The T-NTs were longer than the deposited film thickness, indicating selective electrochemical oxidation/dissolution of Ti by the organic electrolyte. The T-NT did not peel off from the substrate, exhibiting good adhesion even after annealing in air at high temperature. In order to extend the utility of T-NT to make compact and powerful strain gauges [9], sensors [5,6], and drug delivery systems [10], integration of these devices with electronic circuits is required, which in turn is incumbent on a substrate being compatible with the current semiconductor fabrication techniques. With the use of Si as substrate, as typically exemplified in the current semiconductor industry, T-NT-based devices have a great potential for large-scale production and commercialization.

## Methods

### Thin film deposition

Clean n-type (100) Si wafer with resistivity of 1 to 5 Ω cm were used as the substrates for T-NT growth. Wafers were subjected to water vapor-free thermal oxidation at 1,000 °C to form 100 nm of dry SiO_2_. Ti film 650 nm thick was then deposited on the wafer by D.C. sputtering in a Denton Discovery 18 sputter system (Denton, Moorestown, NJ, USA). Ti target of 99.2% to 99.7% purity purchased from Kurt. J. Lesker (East Sussex, UK) was used for sputtering. The sputter chamber was pumped down to pressures < 2 μTorr. The Si substrate was heated to 200 °C, and the same temperature was maintained during sputtering [7]. Argon gas was pumped into the chamber as the plasma source. The argon gas flow was set such that the pressure in the chamber during sputtering is about 2 mTorr. After sputtering, the wafer was cooled down to room temperature in vacuum to minimize the formation of native TiO_2_.

### T-NT synthesis

**Figure 1:**
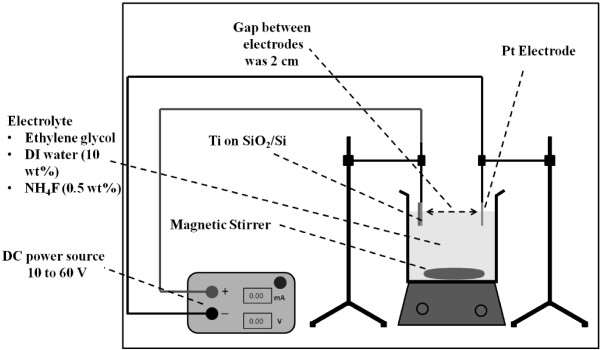
The setup used for the synthesis of T-NT from D.C.-sputtered Ti film on Si wafer.

TiO_2_ nanotubes were synthesized at room temperature via electrochemical anodization. Figure 1 shows the schematic of the experimental setup used for the T-NT synthesis. Si wafer with approximately 650-nm-thin Ti film was diced into 1 × 2-cm-sized pieces. Ethylene glycol (89.5 wt.%, from Fisher Scientific, Waltham, MA, USA), deionized (DI) water (10 wt.%), and ammonium fluoride (0.5 wt.%, from Fisher Scientific) were used as the anodizing electrolyte [11]. The Ti film was degreased with acetone and isopropanol alcohol followed by rinsing in DI water. The Ti film was connected to the positive terminal of the voltage supply via alligator clips to form the anode. The Si is electrically isolated due to the thermally grown SiO_2_, and hence, the Si substrate does not take part in electrochemical reactions that occur during anodization. One-millimeter-thick platinum foil with 1 cm^2^ area was connected to the negative terminal of the voltage source to form the cathode. Polyimide tape was used to mask the non-Ti portion of the diced substrate to prevent any reaction between the electrolyte and the substrate. The gap between the electrodes was 2 cm. Only about 1 cm^2^ of the diced Ti/SiO_2_/Si substrate was subjected to anodization. Magnetic stirring was used to supply fresh electrolyte to the anodization site. A magnetic stirrer 2 cm long at 90 rpm was used to stir about 100 ml of the electrolyte. The rotation rate was chosen so that undesired vibrations due to stirring during anodization were minimum. The anodization voltage was ramped up at the rate of 1 V/s. The anodization was carried out at voltages varying from 10 to 60 V (D.C.). After anodization, the tubes were again cleaned in acetone and isopropanol alcohol followed by rinsing in DI water. The samples were then dried in air. The tubes were annealed in air at 350 °C for 2 h. The temperature was ramped up at a rate of 1.15 °C/min.

### Characterization

Scanning electron microscopy (SEM) micrographs from FEI NanoNova SEM (FEI Co., Hillsboro, OR, USA) were used to characterize the diameter and length of the tubes, along with its structural morphology. Bruker Dimension ICON-PT atomic force microscopy (AFM) micrographs (Bruker AXS, Madison, WI, USA), along with the SEM micrographs, were used to study the effect of anodization time on the tubes’ surface morphology. AFM micrographs using the PeakForce QNM (Bruker AXS) technique were obtained using ultra sharp AFM tips (10 nm diameter). The AFM micrographs were obtained at three different regions, and the average value was used for the roughness. Diffuse-reflectance UV–vis spectroscopy (UV-3600, Shimadzu, Kyoto, Japan) was used to characterize the band gap of the material. X-ray diffraction, utilizing the Philips X’Pert X-ray diffraction (XRD) system (Philips, Amsterdam, Netherlands), with incidence angles ranging from 20° to 80° was used for identification of crystalline phases and determination of stress in the nanotubes.

## Results and discussions

**Figure 2:**
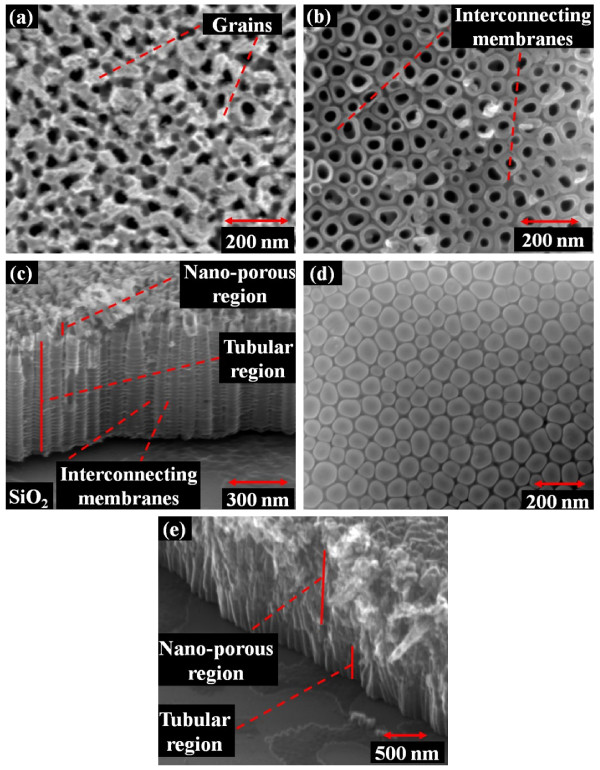
**SEM micrographs.** The T-NT formed by anodization of 650-nm-thick, D.C.-sputtered Ti at 20 V for 1 h. (**a**) Top view of the T-NT. (**b**) Top view after milling about 400 nm of the T-NT top surface. (**c**) Side view showing the length and outer diameter of the tubes (imaged at 60° tilt). (**d**) Bottom of the tube after peeling from the wafer. (**e**) The increase in the nano-porous region due to the presence of thermally induced native TiO_2_ (1-μm-thick film).

SEM micrographs of TiO_2_ nanotubes formed by anodization of sputtered Ti film are shown in Figure 2. From the top-view micrograph (Figure 2a), the surface appears to have a nano-porous/non-tubular structure. As pointed in the figure, pronounced grains of the sputtered film can be seen. Anodization starts at points of high electric field strength (hot spots) formed at grain boundaries. Upon milling the porous portions of the film, tubular-structured tubes can be seen (Figure 2b), and the grains of the sputtered film are not visible. Similarly, from the cross-section micrograph (Figure 2c), non-tubular and the tubular portions of the tubes can be seen. The porous/non-tubular portions consisted of about few tens of nanometer, as pointed in the figure. Self-aligned T-NT arrays interconnected along the sidewalls via thin enclosing membranes can also be observed from the top and side view (pointed in Figure 2b,c). The interconnecting membranes on the side of the nanotubes may be due to the addition of water to the anodizing electrolyte [12]. Figure 2d shows the base of the T-NTs, which was obtained by peeling off from the wafer. The bottom of the tubes show a random distribution of pentagonal and hexagonal patterns, which is also observed when T-NT is synthesized on Ti foils. T-NT growth via electrochemical anodization is the result of Ti oxidation and selective electric-field-assisted chemical etching of oxide. The steps taking place during electrochemical anodization are summarized as follows. Ti reacts with water to form a thin layer of TiO_2_ in the presence of the electric field. TiO_2_ reacts with fluoride ions from ammonium fluoride in the presence of electric field and is selectively etched in a direction defined by the electric field, exposing the underlying metallic Ti to the electrolyte. Again, the exposed Ti metal undergoes oxidation followed by etching. This continues until all the Ti metal is oxidized and selectively etched, resulting in a tubular structure. The synthesis of T-NT from Ti by electrochemical anodization is reviewed in detail in the literature [2]. The formation of the non-tubular nano-porous portion of the T-NT is due to the presence of native TiO_2_ and/or the pronounced grain boundaries. The term native TiO_2_ refers to a thin (10 to 30 nm) TiO_2_ layer formed due to exposure of the sputtered film to atmosphere and is present before subjecting the sample to anodization. Native TiO_2_ prevents the traditional formation of the tubes; however, the electrolyte etches the native TiO_2_ at electric field ‘hot spots’ formed at the pronounced grain boundaries. Upon reaching the metallic Ti, regular tube formation is observed. Similar porous structure formation has been observed in anodizing mechanically polished Ti foil [13] as well as in template-based two-step anodization process [14]. To corroborate that the formation of non-tubular structure was influenced by the oxidized TiO_2_, a control experiment was performed by heating the Ti film in air at 200 °C for 15 min to thicken the TiO_2_ layer, followed by anodization. From the SEM micrographs, a significant increase in the thickness of the non-tubular oxidized layer was observed (Figure 2e). Hence, exposure of the film to air and high temperatures was minimized to reduce native TiO_2_ formation in the T-NT synthesis method. Also, it is noted that the Si substrate or the substrate thickness has no effect on the synthesis of the nanotubes since it is chemically, as well as electrically, isolated during anodization. The sole purpose of the substrate is to provide a mechanical support for the sputtered thin films, and the Ti film was directly biased for anodization. Polyimide tape was used with the purpose of chemically isolating the substrate from the anodizing electrolyte. It was observed that Si would roughen with long exposure to the fluoride ions of the anodization electrolyte (1 h). However, no observable change in the TiO_2_ nanotube formation was observed in the absence of the polyimide tape.

**Figure 3:**
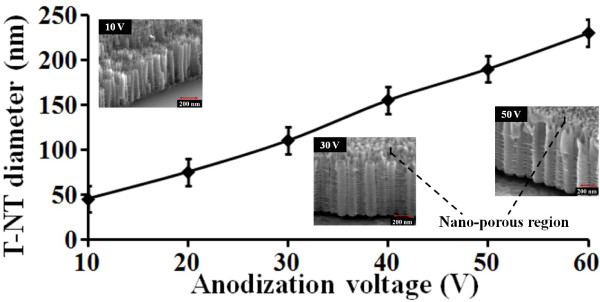
**Change in T-NT diameter with anodization potential after 1 h anodization.** The gap between electrodes was 2 cm. Insets show SEM micrographs of T-NT formed by anodization at 10, 30, and 50 V.

Figure 3 shows the variation in the mode average diameter of the nanotubes with the anodization potential annotated with some inset SEM micrographs of T-NT formed at 10, 30, and 50 V, illustrating the change in the tube diameter/morphology. Ordered tube formation is observed at 10 V and above. However, relatively more uniform/ordered tube formation is observed at 20 V and higher. From the SEM micrographs, it can be seen that with the increase in voltage, the diameter as well as the thickness of the tube walls increases, which is similar to the T-NT synthesized on Ti foil [2]. The nanotubes’ outer diameter increased linearly from about 45 to 230 nm with increase in anodization voltage from 10 to 60 V. The nanotubes formed at all voltages have a non-tubular/nano-porous region, as seen in Figure 2. However, the non-tubular region is relatively thin (10 to 30 nm) and is not clearly visible in the inset side-view SEM micrographs in Figure 3. It was consistently observed that the tube length was about 1.4 times longer than the thickness of the deposited Ti film. The nanotubes were longer than the thickness of the Ti film due to the addition of oxygen atoms during anodization. Similar change in the film thickness due to oxidation can be observed in the case of Si [15]. The tubes did not elongate further irrespective of longer anodization time. Anodization potential also did not have any effect on the length of the tubes. This is because the length of the nanotube is limited by the thickness of the sputtered film, which is the source of the Ti atoms. The nanotube diameter and nanotube formation rate depend on the electric field strength and, hence, changes with anodization potential [2]. However, the length of the tube is mainly dependant on the supply of Ti available for anodization. The top surface of the tubes (few tens of nanometer) had smaller tube diameter compared to the lower portion of the tubes and showed a tapering structure. This may be due to anodization of the film during voltage ramp-up phase [16] and/or the effect of native titanium oxide. No visibly perceptible physical changes were seen after the annealing stage, thus demonstrating the high-temperature stability of the synthesized films.

**Figure 4:**
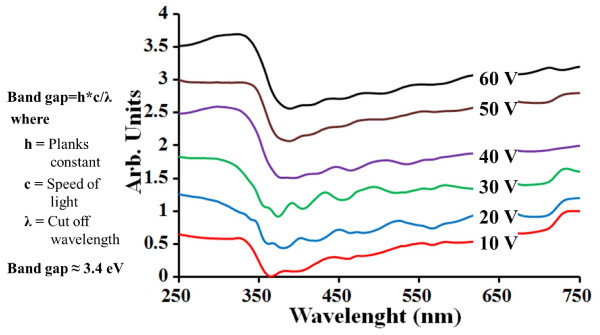
**Diffuse-reflectance UV–vis spectroscopy.** The T-NT on Si after anodization at 10 to 60 V and annealing in air at 350 °C. The anodization time was 1 h. The band gap was about 3.4 to 3.25 eV.

Figure 4 shows the diffuse-reflectance UV–vis spectroscopy of the tubes after annealing. The tubes showed maximum absorbance edge at wavelengths around 365 to 380 nm, corresponding to a band gap of 3.4 to 3.25 eV. One absorption band with increasing visible light absorbance tail may be due to the oxygen vacancies in TiO_2_[17]. The analysis was performed to confirm that the optical absorption characteristic is similar to that of T-NT synthesized on titanium foils [18].

**Figure 5:**
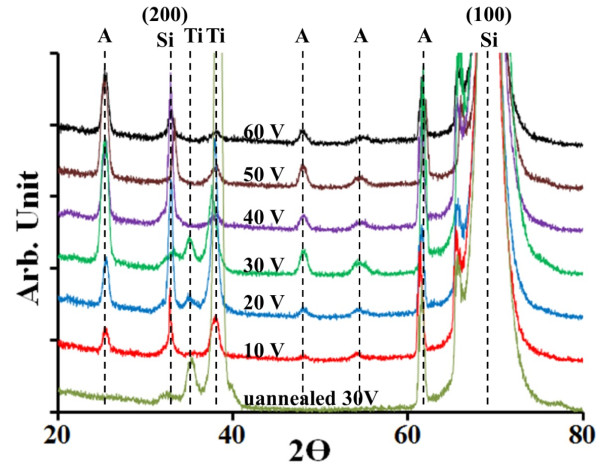
**XRD of the nanotubes before and after annealing in air at 350** °**C for 2 h.** The anodzation time was 1 h. A, anatase form of TiO_2_; Si, silicon substrate; Ti, unanodized Ti.

Figure 5 shows the XRD patterns of the nanotubes before and after annealing. XRD showed that prior to annealing, irrespective of the anodization potential, the tubes were mostly amorphous with the occurrence of high-intensity 2*θ* peaks at 35°, 38° showing the presence of Ti, and a peak at 62° showing traces of anatase TiO_2_, a high-intensity peak at 69° showing the presence of single crystal (100) Si, and a low-intensity peak at 33° indicating (200) Si, which may be due to the stress induced in (100) Si by the sputtered film. Annealed samples showed a strong presence of anatase TiO_2_ with 2*θ* peaks at 25°, 48°, 54°, 55°, and 62° along with low-intensity peaks present at 35° and 38° for traces of Ti metal, which was not anodized. With increase in anodization voltage, a decrease in the Ti peak intensity is observed. Annealing in air supplemented the oxidization of unanodized Ti, which reduced the Ti peak intensity. Annealed samples also showed an increase in intensity of (200) Si peak, which indicates an increase in stress induced in Si primarily due to annealing. X-ray diffraction analysis confirms the formation of TiO_2_ anatase phase, which may be required for certain titania-based application. There was no noticeable stress-induced shifts in the anatase TiO_2_ peaks, confirming stability of the synthesized substrate-based T-NT.

**Figure 6:**
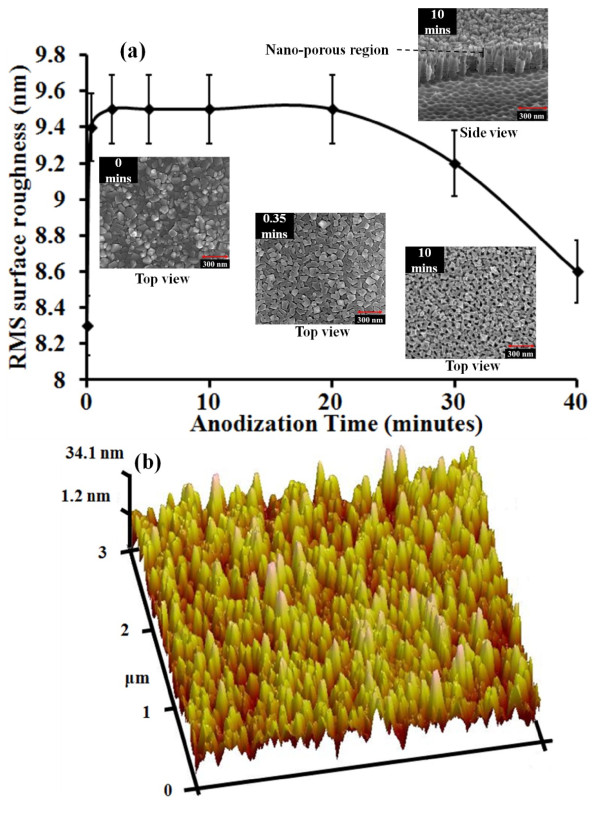
**Change in the root mean square surface roughness of the tubes with anodization time.** (**a**) The samples were anodized at 20 V. Inset SEM micrographs showing the change in surface morphology after anodizing for 0, 0.35, and 10 min. (**b**) AFM image of the Ti film surface after anodization at 20 V for 10 min. The sputtered film thickness was about 650 nm.

The surface of the nanotubes was characterized using AFM and SEM micrographs. Figure 6a shows the change in the root mean square (RMS) roughness of the film surface with anodization time obtained from AFM micrographs. The anodization of the film was carried out at 20 V. Magnetic stirring was used to ensure uniform/faster nanotube formation rate. In the absence of stirring of the electrolyte, the nanotube formation rate would reduce [19,20]. After anodization for 20 s, it can be seen that there is a rapid change in surface roughness as the tubes begin to form, and on further anodization, very little change in surface roughness is observed for up to 20 min. However, from cross-sectional SEM micrographs, it was seen that after 2 min of anodization, formation of regular nanotubes was observed underneath the non-tubular region similar to that seen in Figure 2. After 20 min, the surface roughness decreases. One possible mechanism for this is the pit formation via etching at the grain boundaries because of the occurrence of electric field hot spots at the grain boundaries. After the initial pit formation through the already present native TiO_2_, the electric field is more concentrated at the bottom of the pits, which attracts reactive fluoride ions to the region. This results in etching selectively occurring at the bottom of the pits (high surface roughness during this stage). However, over time, when the nanotube length is considerably long (200 to 400 nm), the availability of the reactive fluoride ions within the tubes is more than what is required for nanotube formation. This results in undesired etching of the tube walls towards the upper region of the nanotubes (resulting in lower surface roughness). Once the Ti film is completely anodized, the nanotubes are electrically isolated from the anode, resulting in the annihilation of electric hot spots across the film. In the absence of the electric field hot spots, slow isotropic etching of the T-NT/native TiO_2_ takes place, which also results in the decline of the RMS surface roughness. The surface roughness is mainly due to the pronounced grains of the sputtered film along with the nano-porous layer. Also, the measurement of the change in the film roughness is limited by the depth up to which the AFM tip can probe. The value provided by the AFM micrographs is only relative value and does not reflect the true roughness of the film. A similar change in the roughness of the film due to anodization can be confirmed from the inset SEM micrographs in Figure 6a. It may also be noted that Figure 6b shows a sample AFM image of the nanotubes’ surface formed by anodizing for 10 min. It is noted that the nanotubes formed have a non-tubular region along with tubular structure similar to the nanotubes seen in Figure 2. However, the non-tubular layer is relatively thin (10 to 30 nm) and is not clearly visible in the inset side-view SEM micrographs. However, from the top-view inset SEM micrographs, nano-porous region similar in morphology to that observed in Figure 2a, which is due to the native TiO_2_ and pronounced surface grains, is seen.

## Conclusions

TiO_2_ nanotube arrays were successfully synthesized on Si wafer by anodization of D.C.-sputtered Ti film in an organic electrolyte. Due to the presence of native titania and pronounced surface grains, formation of nano-porous TiO_2_ layer on top of self-aligned T-NT arrays was observed. From the SEM micrographs of T-NT viewed from the side and those of milled T-NT, the regular formation of nanotubes below the nano-porous layer was deduced. The diameter of the nanotubes varied linearly from 45 to 240 nm with an increase in anodization voltage from 10 to 60 V, respectively. SEM micrographs showed that the length of T-NT was about 1.4 times the thickness of the sputtered Ti film irrespective of the anodization voltage or longer anodization time. XRD characterization showed that unannealed tubes, irrespective of the anodization voltage/time, formed amorphous TiO_2_ nanotubes, and upon annealing in air at 350 °C, anatase phase TiO_2_ nanotubes was formed. AFM and SEM micrographs showed that on complete anodization of the film, the electrolyte etches TiO_2_, resulting in tubes with thinner walls. It can be concluded that the T-NT formed on Si wafer has similar properties to T-NT formed on foil via various characterizations tools, giving rise to potential applications demonstrated by T-NT synthesized on Ti foil. Upon combining the use of well-established semiconductor fabrication techniques used in the semiconductor industry, integration of T-NT in MEMS devices and subsequent large-scale commercialization looks promising.

## Competing interests

The authors declare that they have no competing interests.

## Authors’ contributions

The experiments and characterization presented in this work were carried by KNC. The experiments were designed by KNC and YRS. KNC, YRS, SKM, LWR, PT and MM analyzed and discussed the results obtained from the experiments. The manuscript was prepared by KNC and YRS, and SKM, LWR, PT and MM helped with draft editing. All authors read and approved the final manuscript.
